# Preferential risk of HPV16 for squamous cell carcinoma and of HPV18 for adenocarcinoma of the cervix compared to women with normal cytology in The Netherlands

**DOI:** 10.1038/sj.bjc.6602915

**Published:** 2005-12-13

**Authors:** S Bulk, J Berkhof, N W J Bulkmans, G D Zielinski, L Rozendaal, F J van Kemenade, P J F Snijders, C J L M Meijer

**Affiliations:** 1 Department of Pathology, VU University Medical Center, PO Box 7057, Amsterdam 1007 MB, The Netherlands; 2 Department of Clinical Epidemiology and Biostatistics, VU University Medical Center, PO Box 7057, Amsterdam 1007 MB, The Netherlands

**Keywords:** cervix neoplasm, adenocarcinoma, squamous cell carcinoma, human papillomavirus, epidemiology, cohort study

## Abstract

We present the type-distribution of high-risk human papillomavirus (HPV) types in women with normal cytology (*n*=1467), adenocarcinoma *in situ* (ACIS) (*n*=61), adenocarcinoma (*n*=70), and squamous cell carcinoma (SCC) (*n*=83). Cervical adenocarcinoma and ACIS were significantly more frequently associated with HPV18 (OR_MH_ 15.0; 95% CI 8.6–26.1 and 21.8; 95% CI 11.9–39.8, respectively) than normal cytology. Human papillomavirus16 was only associated with adenocarcinoma and ACIS after exclusion of HPV18-positive cases (OR_MH_ 6.6; 95% CI 2.8–16.0 and 9.4; 95% CI 2.8–31.2, respectively). For SCC, HPV16 prevalence was elevated (OR_MH_ 7.0; 95% CI 3.9–12.4) compared to cases with normal cytology, and HPV18 prevalence was only increased after exclusion of HPV16-positive cases (OR_MH_ 4.3; 95% CI 1.6–11.6). These results suggest that HPV18 is mainly a risk factor for the development of adenocarcinoma whereas HPV16 is associated with both SCC and adenocarcinoma.

Cervical carcinomas are unfortunate complications of longstanding infections with high-risk types of human papillomavirus (hrHPV) (Walboomers *et al*, 1999). Testing for hrHPV types combined with cervical cytology becomes increasingly attractive as data accumulate that a combined test increases the efficacy of cervical screening programmes and triage policies for women with both equivocal and normal cervical smears (Cuzick *et al*, 2003; Khan *et al*, 2005). Possibly, even more efficient screening strategies can be developed by selecting hrHPV types conferring a preferential risk for the development of cervical cancer, and treat these infections more aggressively. In order to assess the preferential risk for cervical cancer and its precursors, type-specific prevalence of hrHPV types in cancer cases should be compared to type-specific prevalence in women without cancer. In a meta-analysis of cervical squamous cell carcinomas (SCCs) compared to high-grade squamous intraepithelial lesions, HPV16, HPV18 and HPV45 appeared to display an elevated prevalence in cervical cancer (Clifford *et al*, 2003a). A second meta-analysis revealed that HPV16 and HPV18 are more prevalent in SCC than in low-grade SIL (Clifford *et al*, 2005). However, a comparison with hrHPV prevalence in women with normal cytology was not made, hampering the translation of these findings to implementation of type-specific hrHPV testing in population-based screening.

Recently in a cross-sectional study, we have demonstrated that among the hrHPV types, HPV16 and HPV33 were significantly more common in women with cervical intraepithelial neoplasia grade 2 or more (⩾CIN2) than in women with normal cytology. However in that study, cases of ⩾CIN2 were retrieved from population-based screening, and consequently, the prevalence of invasive carcinomas as well as adenocarcinoma *in situ* (ACIS) was low.

In order to obtain a more comprehensive view on the change in distribution from hrHPV infections without cytological abnormalities to hrHPV prevalence in cervical cancer, we compared cross-sectional screening data of women with normal cytology to retrospectively collected cases of SCC, adenocarcinoma (AdCx) and ACIS.

## MATERIALS AND METHODS

### Women with normal cytology

Women with normal cytology and a positive hrHPV test were recruited from the POpulation-BAsed SCreening AMsterdam (POBASCAM) trial. The POBASCAM trial is a population-based randomised, controlled trial to evaluate the efficacy of screening using hrHPV testing in conjunction with conventional cytology *vs* cervical screening with conventional cytology only. This trial was conducted within the regular Dutch cervical screening programme in which women aged 30–60 years are screened cytologically with a screening interval of 5 years (van Ballegooijen and Hermens, 2000). In the POBASCAM trial, details of which have been described elsewhere, conventional smears were taken using either a Cervex brush or a cytobrush (Bulkmans *et al*, 2004). After taking the smear, the brush was placed in a vial containing collection medium for hrHPV testing. Cervical smears were then classified according to the Dutch CISOE-A classification, which can be translated to the Bethesda system (Solomon *et al*, 2002; Bulk *et al*, 2004).

Detection of hrHPV was performed by GP 5+/6+ PCR enzyme immunoassay, using a cocktail of 14 high-risk types, that is, HPV16, HPV18, HPV31, HPV33, HPV35, HPV39, HPV45, HPV51, HPV52, HPV56, HPV58, HPV59, HPV66 and HPV68 (Jacobs *et al*, 1997). All hrHPV-positive samples were typed by reverse line blotting (van den Brule *et al*, 2002).

Between 1998 and 2002, a total of 44 102 women were included in the study. Of these women, 3.7% (*n*=1576) were diagnosed with a normal Pap test and a positive hrHPV test. Women who were positive for hrHPV by GP5+/6+−PCR but negative for reverse line blot were excluded (*n*=109), leaving 1467 women for the analysis. All technicians were blinded to hrHPV status and cytological diagnosis of the samples.

### Cases with cervical carcinoma (*in situ*)

All cases of cervical carcinoma (*in situ*) were identified during the course of previous studies (Zielinski *et al*, 2001, 2003). Cases with ACIS and invasive AdCx of the cervix were enrolled from four pathology departments in The Netherlands. In the period 1996–2000, formalin-fixed specimens of 62 ACIS cases and 77 cases of AdCx were identified that were adequate for hrHPV PCR analysis. Cases with SCC were identified in a geographically defined region of The Netherlands. Archival formalin-fixed material was collected of 91 SCC cases from the period 1981 to 1998.

HrHPV detection was performed by GP5+/6+ PCR as described above. In addition, all GP5+/6+ PCR-negative samples were evaluated by E7 region type-specific PCR for the same 14 high-risk types in order to exclude that cases with hrHPV DNA integrated in the GP5+/6+ primer binding region were diagnosed as hrHPV negative (Walboomers *et al*, 1999). Laboratory personnel unaware of the histological diagnosis performed hrHPV determinations.

Of the cases with ACIS, one tested negative for hrHPV by both PCR tests, and four cases tested positive only by type-specific E7 PCR. Seven of the 77 AdCx cases tested negative for hrHPV by both tests, and seven tested positive only for type-specific E7 PCR. Of the women with SCC, seven cases tested negative for hrHPV by both assays and one case tested positive by type-specific E7 PCR only, leaving 83 cases with SCC for analysis.

### Statistical analysis

Differences in prevalence of hrHPV types for women with normal cytology compared to women with cervical carcinoma were examined using the Mantel–Haenszel common odds ratio (OR_MH_), 95% Confidence intervals (95% CI's) were calculated. Statistical significance was tested by Cochran's Mantel–Haenszel test. Only OR_MH_ with values of 1.0 or higher are reported. Data were adjusted in 5-year age categories (i.e., below 29 years, 29–33, 34–38, 39–43, 44–48, 49–53, 54–58, 59–61, and 61 years and over), matching with age categories in nationwide screening. The presence of an association between OR_MH_ and age was tested by Breslow–Day's test of homogeneity. Variations in the prevalence of single and multiple infections were assessed using *χ*^2^ analysis. Analyses were performed separately for women with either single and multiple infections or single infections only. Since HPV18 prevalence heavily dominated in AdCx and ACIS, and HPV16 infections dominated in both normal cytology and SCC, we also calculated risk estimates after either discarding HPV16-positive cases, or HPV18-positive cases, or both.

A potential source of bias may be that women with normal cytology were matched with cases with cancer in the same 5-year age category. As it takes at least 8–10 years to develop invasive carcinoma of the cervix after infection with hrHPV, we repeated the analyses matching cases with cancer with normal controls 10 years younger (van Oortmarssen and Habbema, 1995; Zielinski *et al*, 2001; Berkhof *et al*, 2005). For these analyses, we included women with normal cytology who were sampled during the enrolment phase of the POBASCAM trial, but did not meet the age criteria for population-based cervical screening (*n*=58).

### Role of the funding source

The sponsor of the study had no role in study design, data collection, data analysis, data interpretation, or writing the report.

## RESULTS

### Study subjects

The mean age of women with normal cytology was 37 years (range 17–63 years). The mean age of women with ACIS was 37.1 years (range 23–55) and did not differ statistically from the age of women with normal cytology. Women with invasive AdCx and SCC had a mean age of 44.7 years (range 28–79 years) and 49 years (range 27–88 years), respectively. Both for women with AdCx (*P*<0.01) and SCC (*P*<0.01) the mean age was statistically significantly increased compared to women with normal cytology or ACIS.

Of the 1525 women with normal cytology, 268 (17.6%) were diagnosed with a multiple hrHPV infection. Multiple infections were also common in women with ACIS (13/61, 21.3%). Women with invasive AdCx (5/70, 7.1%) and SCC (9/83, 10.8%) had a lower prevalence of multiple hrHPV infections than women with either normal cytology or a noninvasive lesion (*P*=0.007).

### HrHPV type-specific prevalence in women with normal cytology

Table 1 displays the hrHPV type-specific prevalence in women with normal cytology compared with cases of cervical cancer (*in situ*). In women with normal cytology, HPV16 accounted for 30.0% of hrHPV positivity and HPV31 for 15.7%. Another 10.0% of hrHPV infections in women with normal cytology involved HPV18 and 8.0% HPV45. Type-specific prevalence did not substantially change when analysing women with single infections separately (Table 1).

### HrHPV type-specific prevalence in women with cervical ACIS

In women with ACIS, HPV18 infections (66.2%) dominated. Of the remaining hrHPV infections, HPV45 accounted for 6.2% of infections, HPV16 for 33.8%, and HPV31 for 4.6% (Table 1). No other single hrHPV infections were observed in ACIS. When cases with single infections were analysed separately, type-specific prevalence did not change substantially.

When analysing for individual types, women with ACIS were statistically significantly more likely to carry HPV18 than women with normal cytology (OR_MH_ 21.8; 95% CI 11.9–39.8). The prevalence of HPV16 was comparable between women with normal cytology and women with ACIS. However, after excluding HPV18-positive cases from the analyses, prevalence of HPV16 was 9.4 times as frequently present in cases with ACIS compared to women with normal cytology (95% CI: 2.8–31.2). After excluding both HPV16- and HPV18-positive cases, prevalence of HPV45 was also statistically significantly increased in cases with ACIS (OR_MH_ 14.0; 1.3–150.9). When cases with single infections were analysed separately, type-specific prevalence did not change substantially. All analyses were repeated when matching ACIS cases with normal controls 10 years younger, but estimates were not affected (data not shown). For none of the HPV types, OR varied with age (data not shown).

### HrHPV type-specific prevalence in women with cervical adenocarcinoma

In women with AdCx, HPV18 infections (57.1%) displayed the highest prevalence. Of the remaining hrHPV infections, HPV45 accounted for 11.4% of infections and HPV16 for 35.7% (Table 1). When cases with single infections were analysed separately, type-specific prevalence did not change substantially.

Results were comparable for women with ACIS and for women with invasive AdCx since HPV16 prevalence was not different between AdCx and normal cytology (OR_MH_ 1.3; 95% CI 0.8–2.2) and women having AdCx were more likely to be infected by HPV18 than women with normal cytology (OR_MH_ 15.0; 95% CI 8.6–26.1). After exclusion of HPV18-positive cases, both HPV16 and HPV45 were statistically significantly associated with AdCx (OR_MH_ 6.6; 95% CI 2.8–16.0 and OR_MH_ 4.3; 95% CI 1.7–10.6, respectively). Results for single infections only were comparable (Table 1). All analyses were repeated matching cases with AdCx with normal controls 10 years younger, but estimates were not affected (data not shown). For none of the HPV types, OR varied with age (data not shown).

### HrHPV type-specific prevalence in women with SCC

Compared with cervical AdCx and its precursor ACIS, results were reversed for HPV16 and HPV18 in women with SCC. Women with SCC had an increased prevalence of HPV16 infections (69.9%) compared to HPV18 infections (12.0%). Compared to the cases with cervical AdCx, SCC showed more diversity in types as only HPV51, HPV52, HPV59, HPV66, and HPV68 did not occur at all in the cases of SCC.

Women having SCC were significantly more likely to carry HPV16 than women with normal cytology (OR_MH_ 7.0; 95% CI 3.9–12.4). Since HPV16 dominated in cases of SCC, analyses were repeated after exclusion of HPV16. In these analyses women with SCC were more likely to carry HPV18 infections than women with normal cytology (OR_MH_ 4.3; 95% CI 1.6–11.6). Again, we investigated whether less prevalent types displayed type-specific increases in prevalence as well by excluding both HPV16 and HPV18 from the analyses. In multiple infections, both HPV 31 and HPV39 were more frequently present in SCC than in normal cytology after exclusion of both HPV16 and HPV18 coinfections (OR_MH_ 3.5; 95% CI 1.0–11.5, and OR_MH_ 5.4; 95% CI 1.3–21.7, respectively). However, the prevalence of these types was not increased in single infections. Results for single infections were comparable. After matching SCC cases with controls 10 years younger, none of the estimates were affected appreciably (data not shown). For none of the HPV types OR varied with age (data not shown).

## DISCUSSION

This study on the distribution of 14 hrHPV types revealed marked differences between cervical adenocarcinoma and its precursors, and SCC. The prevalence of HPV16 is increased in SCC compared to normal cytology, whereas HPV18 is more prevalent in adenocarcinoma and its precursor. However, when accounting for the distorting effect of extremely high prevalence types, HPV16 and HPV45 are also associated with adenocarcinoma and its precursors, and HPV18 is associated with SCC as well. These data suggest that within the group of high-risk types of which the association with cervical cancer has already been established beyond any doubt (Munoz *et al*, 2003), infections with either HPV16, HPV18, or HPV45 confer a preferential risk to develop a malignancy of the uterine cervix.

Some methodological aspects of this study need to be discussed. Firstly, we compared cross-sectional data of women with normal cytology obtained from the POBASCAM trial with retrospectively collected cases of cervical cancer. This approach may have biased our estimates of risk associated with specific hrHPV types. Women with cervical cancer were on average older than the women with normal cytology, and age has been shown to be associated with hrHPV type-specific prevalence (Castle *et al*, 2005a). We used two methods for to correct for the age difference. Firstly, we analysed our data stratified in age categories. Secondly, a potential source of bias may be that it takes at least 8–10 years to develop invasive carcinoma of the cervix (van Oortmarssen and Habbema, 1995; Zielinski *et al*, 2001). Women with cancer may have contracted an hrHPV infection 10 years before they were diagnosed with cancer. We repeated the analyses matching cases of cancer with normal controls 10 years younger, and our estimates were not affected, indicating the robustness of our data. Also, we defined women with normal cytology as women having a screening smear diagnosed as normal without either histologically or cytologically diagnosed lesions in the 2 years preceding the screening smear. In the Dutch screening programme, these women are considered to be free of cervical disease, and they will not be called for cervical screening for the next 5 years (van Ballegooijen and Hermens, 2000). However, our population with normal cytology may have contained a small number of women with either an underlying high-grade lesion or who may develop a high-grade lesion during follow-up. This diagnostic bias would have a diluting effect on the risk estimates obtained by our study, as hrHPV prevalence in the normal population would have resembled the cervical lesion cases more closely. A strong point of our cross-sectional approach is that we were able to use a reference group of women with normal cytology taken from the same geographic region as cases with cervical cancer. In contrast, other studies relating hrHPV prevalence to histological type have relied on pooling of data obtained from worldwide studies to provide estimates of hrHPV prevalence in cancer and its precursor stages and regional variations in type-specific prevalence may have affected comparisons in these studies (Clifford *et al*, 2003a, 2003b, 2005).

Thirdly, we have performed a type-specific E7 PCR in women with carcinoma in order to diagnose integrated hrHPV infections, whereas women with normal cytology were not evaluated using the type-specific PCR. This may have resulted in a confirmation bias in the diagnosis of hrHPV infections, as integrated hrHPV infections in women with normal cytology may not have been diagnosed. However, integration of the hrHPV occurs late in the progression from normal epithelium to carcinoma, and viral integration is extremely rare in a population of women with normal cytology (Munoz *et al*, 2003). Therefore, we do not consider our approach biased.

In addition to other studies demonstrating that adenocarcinomas are more often HPV18 positive than HPV16 positive (Pilch *et al*, 2001; Schwartz *et al*, 2001; Altekruse *et al*, 2003; Burk *et al*, 2003; Clifford *et al*, 2003b; Huang *et al*, 2004), we have now shown that when comparing invasive adenocarcinoma cases to cytologically normal controls, the OR_MH_ is only 1.3 for HPV16 and 15.0 for HPV18. Conversely, when comparing invasive SCC cases to normal controls, OR_MH_ is 7.0 for HPV16 and only 1.3 for HPV18. This suggests that HPV16 and HPV18 are associated with a preferential risk compared to the other high-risk types of hrHPV for the development of either SCC or adenocarcinoma. Combining our analyses both including HPV18 and excluding HPV18, our data suggest that HPV18 is mainly a risk factor for the development of adenocarcinoma whereas the highly aggressive HPV16 is associated with both SCC and adenocarcinoma. Alternatively, HPV16 and HPV18 might preferentially induce differentiation in either squamous or columnar direction respectively after infection of epithelial stem cells localised in the basal layer of the epithelium. The hrHPV types tested for in this study other than HPV16, HPV18, and HPV45, did not reveal an increased prevalence in either histological subtype of cervical cancer, suggesting that the other types all pose a similar relatively low risk of progression to cancer. This also includes HPV33, which is prevalent in lesions ⩾CIN2 (Bulkmans *et al*, 2005). However, a plausible explanation might be that HPV33 has the potential to induce high-grade CIN lesions relatively easy, whereas its capacity to induce progression from high-grade CIN to invasive carcinoma might be relatively low (Bulkmans *et al*, 2005).

What are the consequences of our findings for cervical screening? Recently, it was shown that both HPV16 and HPV18 infections in women with normal cytology are associated with an increased 10-year absolute risk for high-grade lesions and cervical cancer (Khan *et al*, 2005). However, two other recently published studies did not demonstrate an association of HPV18 with cytological abnormalities and high-grade histological lesions in either short-term follow-up or in a cross-sectional design (Bulkmans *et al*, 2005; Castle *et al*, 2005b). These data suggest that HPV18 infections, which we have shown to be preferentially increased in prevalence in cervical adenocarcinomas, either do not induce cervical lesions diagnosed as abnormal cytologically or that cervical lesions associated with HPV18 are not diagnosed as a result of sampling error, since these lesions are more often localised high in the endocervical canal (Woodman *et al*, 2003). Whatever the cause, our results show that HPV18 has a preferential risk for AdCx and ACIS. Being aware of this association should warrant a less expectant attitude for women with persistent HPV18 infections and normal cytology to refer women to colposcopically mediated biopsies and endocervical sampling in case of a normal transformation zone.

In conclusion, we have shown that HPV16, HPV18, and HPV45 display an increased prevalence in cervical cancer compared to cytologically normal smears. HPV16 confers the greatest risk for SCC and HPV18 for adenocarcinoma of the cervix. These data strongly argue for hrHPV type-specific risk stratification of women with normal cytology and a positive hrHPV test participating in cervical screening programmes.

### Ethics approval

The study was approved by the Medical Ethics Committee of the VU University Medical Center, Amsterdam, The Netherlands (nr 96/103), and by the Ministry of Public Health, The Hague, The Netherlands (VWS nr 328 650).

### POBASCAM study collaborators other than authors

Dr K v Groningen (Spaarne Ziekenhuis, Heemstede, The Netherlands), Dr W Ruitinga (Stichting PA Laboratorium Kennemerland, Haarlem, The Netherlands), Dr ME Boon (Leiden Cytology and Pathology Laboratory, Leiden, The Netherlands), Dr M van Ballegooijen (Department of Public Health and Social Medicine, Erasmus University Rotterdam, The Netherlands), Dr AJP Boeke (Institute for Research in Extramural Medicine, VU University Medical Center, Amsterdam, The Netherlands), Prof Dr RHM Verheijen (Department of Obstetrics and Gynaecology, VU University Medical Center, Amsterdam, The Netherlands).

## Figures and Tables

**Table 1:** HrHPV type-specific prevalence in adenocarcinoma and its precursors and squamous cell carcinoma *vs* normal cytology - single infections and single and multiple infections combined

**Type**	**Normal *N*=1467**	**ACIS *N*=65**	**AdCx *N*=70**	**SCC *N*=83**	**Normal vs ACIS**	**Normal vs AdCx**	**Normal vs SCC**
*Multiple* a	*N* (%)	*N* (%)	*N* (%)	*N* (%)			
16	440 (30.0)	22 (33.8)	25 (35.7)	58 (69.9)	1.3 (0.7–2.2)	1.3 (0.8–2.2)	7.0 (3.9–12.4)
18	146 (10.0)	43 (66.2)	40 (57.1)	10 (12.0)	21.8 (11.9–39.8)	15.0 (8.6–26.1)	1.3 (0.6–2.8)
31	230 (15.7)	3 (4.6)	—	6 (7.2)	—	—	—
33	88 (6.0)	—	—	3 (3.6)	—	—	—
35	74 (5.0)	—	—	2 (2.4)	—	—	—
39	70 (4.8)	—	1 (1.4)	4 (4.8)	—	—	1.5 (0.5–4.3)
45	118 (8.0)	4 (6.2)	8 (11.4)	5 (6.0)	—	1.6 (0.8–3.5)	—
51	98 (6.7)	1 (1.5)	—	—	—	—	—
52	92 (6.3)	—	1 (1.4)	—	—	—	—
56	134 (9.1)	—	—	2 (2.4)	—	—	—
58	93 (6.3)	—	—	2 (2.4)	—	—	—
59	43 (2.9)	—	—	—	—	—	—
66	100 (6.8)	1 (1.5)	—	—	—	—	—
68	24 (1.6)	—	—	—	—	—	—

*Single*	*N*=1221	*N*=48	*N*=65	*N*=74			
16	344 (28.2)	14 (29.2)	22 (33.8)	55 (74.3)	1.0 (0.5–1.9)	1.3 (0.7–2.2)	9.2 (4.9–17.3)
18	108 (8.8)	31 (64.6)	36 (55.4)	8 (10.8)	69.4 (20.8–231.8)	15.8 (8.8–28.4)	1.5 (0.6–3.3)
31	170 (13.9)	1 (2.1)	—	3 (4.1)	—	—	—
33	66 (5.4)	—	—	1 (1.4)	—	—	—
35	48 (3.9)	—	—	—	—	—	—
39	37 (3.0)	—	1 (1.5)	1 (1.4)	—	—	—
45	81 (6.6)	2 (4.2)	6 (9.2)	4 (5.4)	—	1.5 (0.6–3.2)	—
51	60 (4.9)	—	—	—	—	—	—
52	57 (4.7)	—	—	—	—	—	—
56	91 (7.5)	—	—	—	—	—	—
58	60 (4.9)	—	—	1 (1.4)	—	—	—
59	31 (2.5)	—	—	—	—	—	—
66	59 (4.8)	—	—	—	—	—	—
68	9 (0.7)	—	—	—	—	—	—

Normal indicates normal cytology; ACIS indicates adenocarcinoma *in situ*; AdCx indicates adenocarcinoma; SCC indicates squamous cell carcinoma.

Analyses are adjusted for age in 5-year strata.

a Multiple and single infections combined.
